# A simple high throughput assay to evaluate water consumption in the fruit fly

**DOI:** 10.1038/s41598-017-16849-6

**Published:** 2017-12-01

**Authors:** Man-Tat Lau, Yong Qi Lin, Stefan Kisling, James Cotterell, Yana A. Wilson, Qiao-Ping Wang, Thang M. Khuong, Noman Bakhshi, Tiffany A. Cole, Lisa J. Oyston, Adam R. Cole, G. Gregory Neely

**Affiliations:** 1 The Dr. John and Anne Chong Lab for Functional Genomics, Charles Perkins Centre and School of Life & Environmental Sciences, The University of Sydney, Sydney, NSW 2006 Australia; 2 Neuroscience Division, Garvan Institute of Medical Research, 384 Victoria Street, Darlinghurst, Sydney, NSW 2010 Australia; 3 Sacred Heart College, Retreat Rd, Newtown, Geelong, Victoria, 3220 Australia

## Abstract

Water intake is essential for survival and thus under strong regulation. Here, we describe a simple high throughput system to monitor water intake over time in *Drosophila*. The design of the assay involves dehydrating fly food and then adding water back separately so flies either eat or drink. Water consumption is then evaluated by weighing the water vessel and comparing this back to an evaporation control. Our system is high throughput, does not require animals to be artificially dehydrated, and is simple both in design and implementation. Initial characterisation of homeostatic water consumption shows high reproducibility between biological replicates in a variety of experimental conditions. Water consumption was dependent on ambient temperature and humidity and was equal between sexes when corrected for mass. By combining this system with the *Drosophila* genetics tools, we could confirm a role for *ppk28* and *DopR1* in promoting water consumption, and through functional investigation of RNAseq data from dehydrated animals, we found *DopR1* expression in the mushroom body was sufficient to drive consumption and enhance water taste sensitivity. Together, we provide a simple high throughput water consumption assay that can be used to dissect the cellular and molecular machinery regulating water homeostasis in *Drosophila*.

## Introduction

Maintaining proper osmolarity is essential for life, and thus is under strong regulation. Internal water homeostasis can be monitored by tissue or cellular osmolarity^1,2^ or osmolarity/fluid volume within the circulatory system^3,4^. Mammals monitor internal water both through peripheral sensors in the kidney^5^ and central receptors in the brain^6^. Body osmolarity can then be regulated through excretion/retention or consumption of water. Water intake is regulated through actions in the hypothalamus^7^ and lamina terminalis^6^, and a well characterised endocrine system involving renin/angiotensin^8^, which promote thirst, and vasopressin^9^, which drives water retention. Dysregulation of internal water can result in dehydration and hypernatremia (high sodium in the blood), leading to low blood pressure, cognitive impairment, and in serious instances kidney injury, intracranial haemorrhage, and death^10^. As such water seeking and consumption is a robust innate behaviour worth systematic cellular and molecular interrogation.

Much progress has been made characterising the receptors and circuitry regulating water consumption in the fruit fly *Drosophila melanogaster*. Early work by the Benzer lab described a robust humidity-seeking behaviour (hygrosensation) in the fly, mediated through a humidity sensor in the arista (on the antenna) and dependent on TRP channels^11^ and ionotropic glutamate receptors^12,13^. When in closer proximity, insects can taste water^14^. In the fruit fly, water taste and consumption is mediated by gustatory^15^ and higher order interoceptive subesophageal zone^16^ neurons and through the water taste receptor *ppk28* ^17,18^. Water homeostasis is further regulated in the hindgut through the osmolyte transporter *inebriated*, which promotes water absorption when animals are fed salty food^19^. Dehydration also enhances water taste sensitivity^20^ and promotes water sensing and drinking behaviour, and in thirsty animals water can be used as a reward for conditioned learning tasks^21^.

Multiple assay systems have been developed to evaluate water sensation or consumption in the fruit fly. Hygrosensation is tested by providing flies with a choice between humid and dry air, and unchallenged animals prefer a low humidity environment^22^. Water taste can be assessed using Ca^2+^ imaging or electrophysiology^15,17,18,23^, by measuring time spent drinking^17^ or water responses using the proboscis extension response^15^. However, these assays are labour-intensive or require specific skills and equipment. Actual water consumption has been measured by spiking water with food coloring^20,21^, however this technique requires animals to be dehydrated before testing and cannot be used to monitor water consumption over long periods. To address these issues, here we present a simple, high-throughput method for assessing *Drosophila* water consumption over time.

## Results

**Figure 1:**
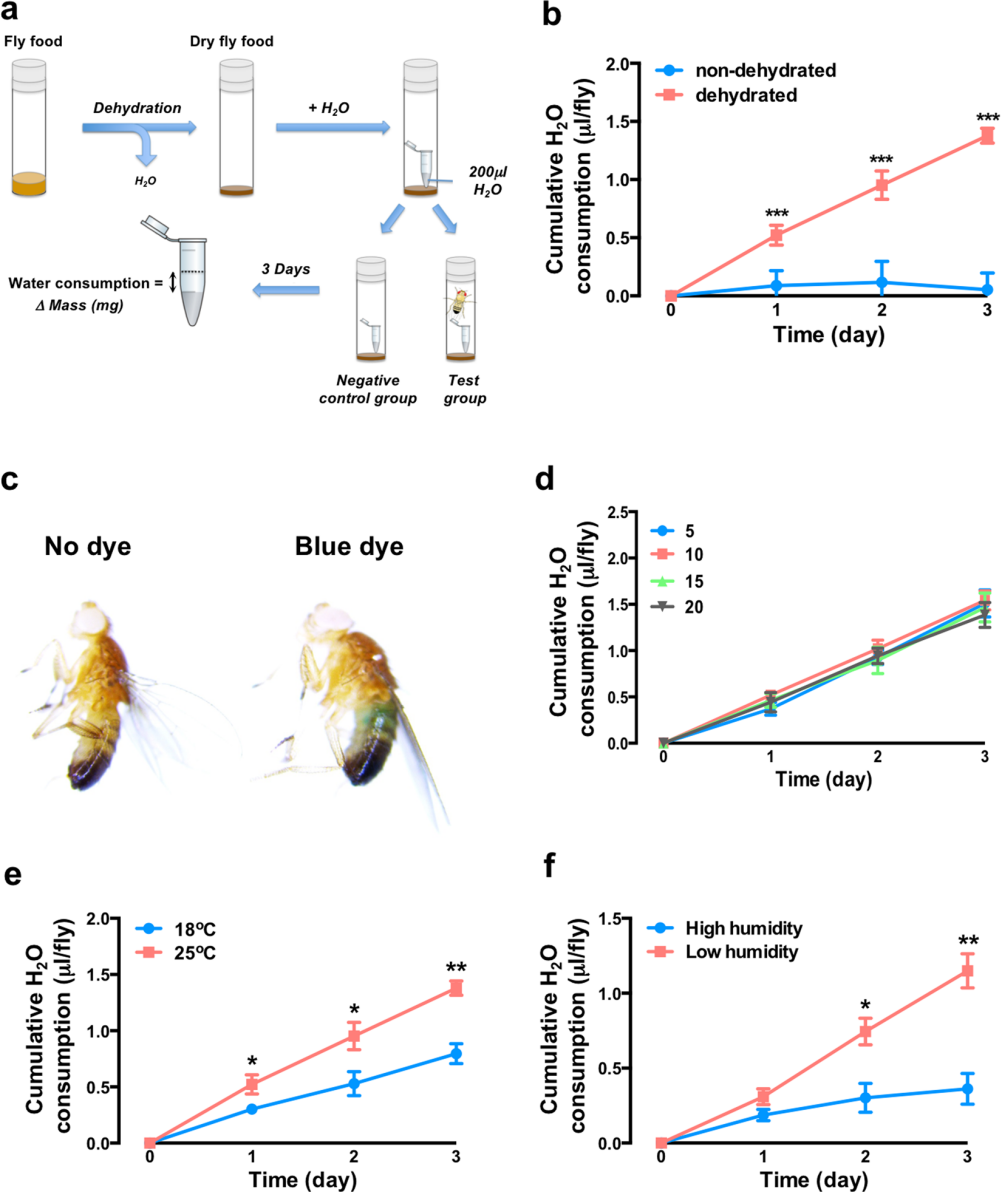
Water consumption assay. (**a**) Schematic diagram of assay. (**b**) Water consumption using non-dehydrated and dehydrated food. (**c**) To facilitate visualisation of consumed water, blue dye has been added to the water and can be seen in the abdomen of the fly. (**d**) The number of flies per chamber does not influence individual water consumption. Five, ten, fifteen, or twenty files were tested per assay for 3 days. (**e**) Temperature regulates water consumption in flies. The water consumption assay was tested in either 18 °C or 25 °C conditions for 3 days. (**f**) Desiccation stimulates water consumption in flies. The water consumption assay was tested in either low humidity (55%) or high humidity (70%) conditions for 3 days. All data represented mean ± S.E.M (n = 3–6). Student’s *t*-test, **p < 0.01; ***p < 0.001; n.s., not significant.

To facilitate the cellular and molecular dissection of thirst, we developed a novel behavioural assay to assess water intake in the fruit fly, *Drosophila melanogaster*. In laboratory conditions, flies obtain their water from food and ambient humidity. To monitor water intake, we separated fly food from water by dehydrating the food and added the water back in a separate receptacle filled with 200 μl of water. Flies are then added to the vial, with a parallel vial left empty as an evaporation control (Fig. 1a). Flies exposed to standard hydrated fly food did not consume the additional water, whereas flies housed in dehydrated food consumed water in the tube at a constant rate over time (Fig. 1b). Water consumption was confirmed by spiking water with blue dye, which could then be observed in the fly abdomen (Fig. 1c). We found fly number per vial had no effect on cumulative water consumption per fly in the range from 5 to 20 flies per vial (Fig. 1d). We observed a temperature-dependence in water consumption, with flies exposed to 25 °C drinking significantly more water than flies housed at 18 °C (Fig. 1e). This response was also dependent on humidity (Fig. 1f), with high humidity conditions (70%) suppressing drinking behaviour whereas lower humidity conditions (55%) promoted drinking behaviour.

**Figure 2:**
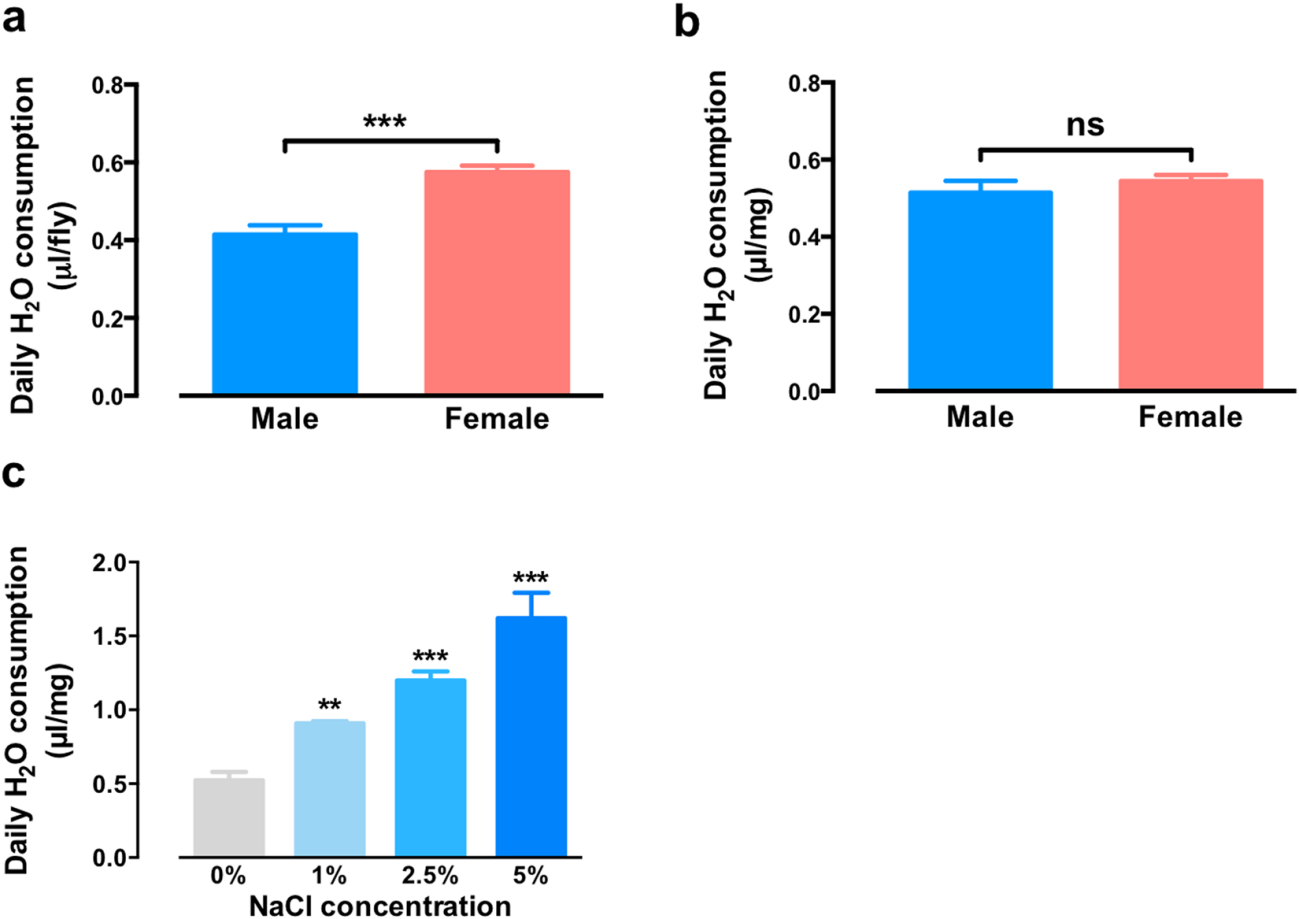
(**a**) Daily water consumption in male and female flies without body weight normalisation. (**b**) Daily water consumption in male and female flies with body weight normalisation. (**c**) Salt stimulates water consumption in flies. All data represented mean ± S.E.M (n = 3–6). Student’s *t*-test, **p < 0.01; ***p < 0.001; n.s., not significant.

We observed a sex-specific difference in drinking behaviour, with females consuming significantly more water than males per fly (Fig. 2a). However, there was no significant difference between sexes once drinking behaviour was normalised for body mass (Fig. 2b). Since variations in fly mass influences intake, we use µl/mg H_2_O consumption for subsequent measurements. Overall, we found steady state water consumption was 65% of total body weight per day. Homeostatic water consumption was not maximal, since inclusion of sodium chloride (NaCl) in the desiccated food could promote increased water consumption in a dose-dependent manner (Fig. 2c). Further, the strong response to salt levels suggests this assay system may also be suitable for rapid functional genetic dissection of the fly renal system^24^. Thus, we report a robust high-throughput assay system for monitoring water intake that can be used to study neurological and renal regulation of water homeostasis in *Drosophila*.

**Figure 3:**
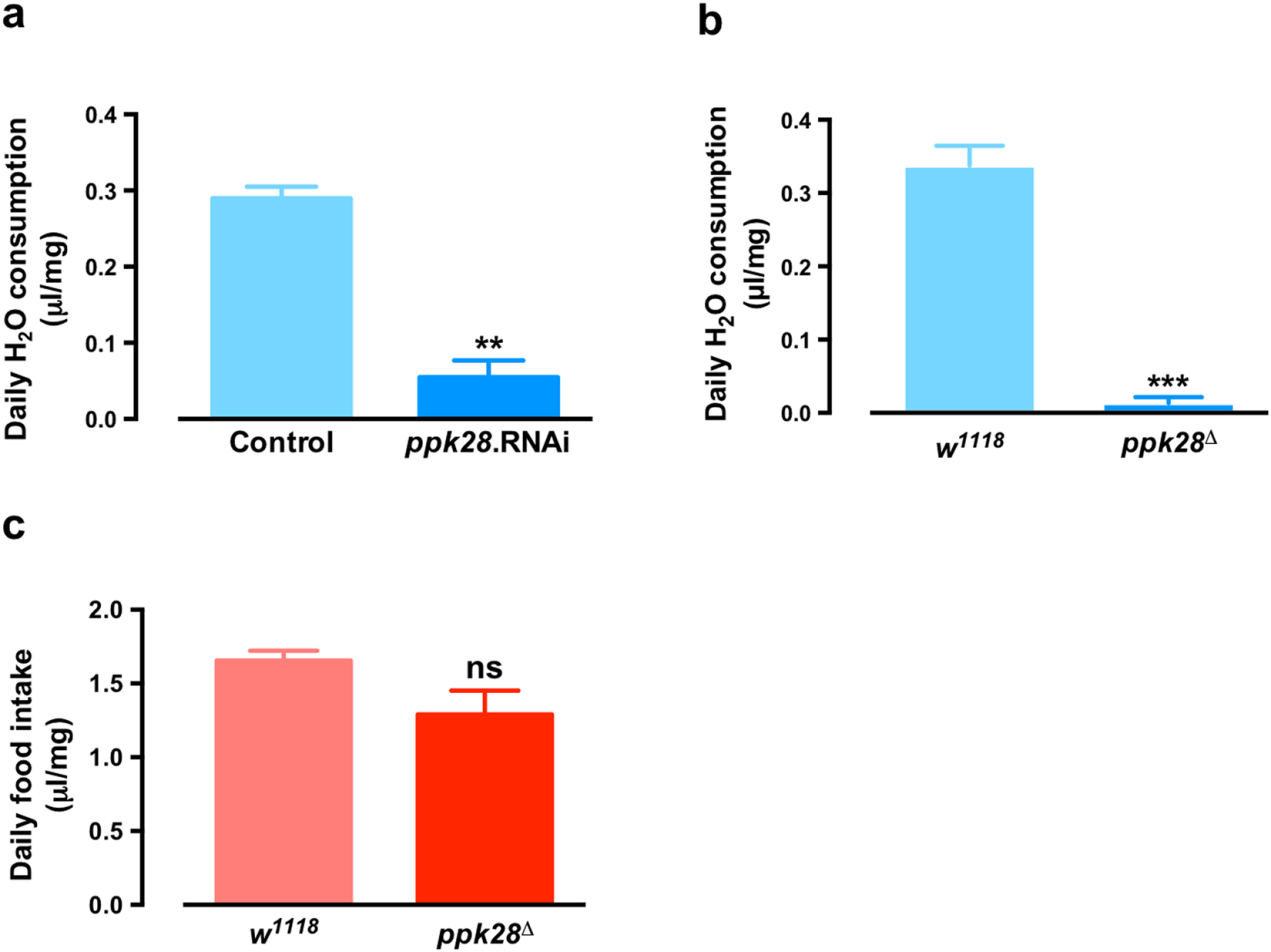
The water taste receptor *ppk28* regulates water consumption. (**a**) Water consumption of control (*elav-Gal4 > w* ^1118^) and *elav-Gal4 > ppk28* RNAi flies. (**b**) Water consumption and (**c**) food intake of *w* ^1118^ control and *ppk28* mutant flies (*ppk28* ^*Δ*^). All data represented mean ± S.E.M (n = 3–6). Student’s *t*-test, **p < 0.01; ***p < 0.001; n.s., not significant.

Flies can taste water specifically, and this occurs via the *ppk28* ^17,18^. *ppk28* was also required for water consumption in our system, with neural specific *elav-Gal4* > *ppk28* RNAi showing a drastic reduction in water consumption compared to *elav-Gal4* > *w* ^1118^ control (Fig. 3a). *ppk28* mutant flies also showed a drastic reduction in water consumption (Fig. 3b) indicating that *ppk28*-dependent water taste is also required for drinking behaviour. The *ppk28*-dependent defect was water-specific, since control and *ppk28* mutant animals showed comparable food intake (Fig. 3c). Thus the *ppk28* water taste receptor is also required for water consumption in this system.

**Figure 4:**
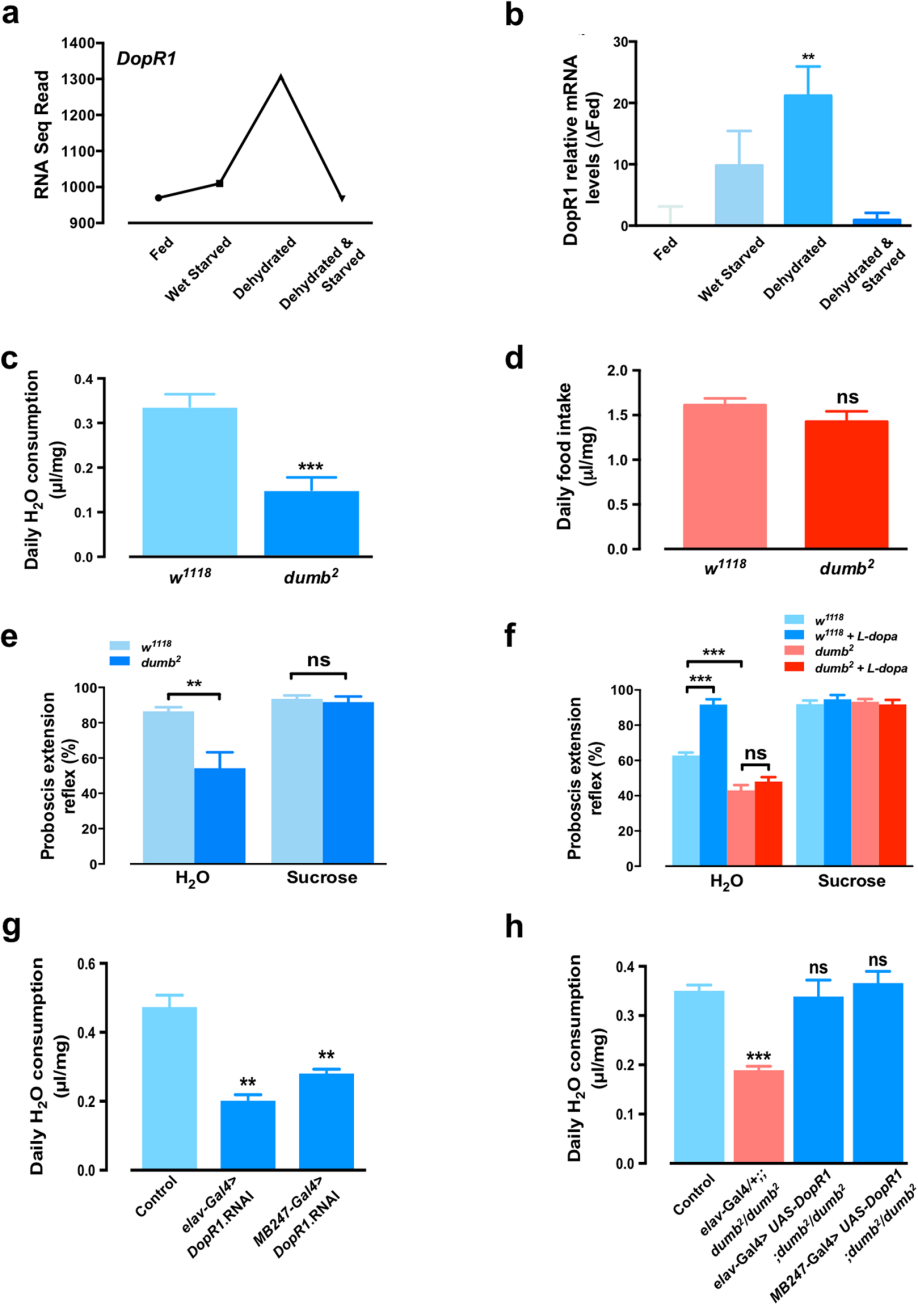
The dopamine receptor *DopR1* regulates water consumption. (**a**) RNA Seq reads of *DopR1* transcripts obtained from the heads of dehydrated or control flies. (**b**) *DopR1* mRNA levels were analysed by RT-qPCR. (**c**) Water consumption of *w* ^1118^ control and *DopR1* mutant flies (*dumb* ^2^). (**d**) Food consumption of *w* ^1118^ control and *DopR1* mutant flies (*dumb* ^2^) highlights a specific defect in water consumption. (**e**) Proboscis extension response (PER) of *w* ^1118^ control and *DopR1* mutant flies to H_2_O and 5% sucrose solution (**f**) L-dopa (3 mg/ml) pre-treatment increases the PER response to water in *w* ^1118^ control but not *DopR1* mutant flies. Sucrose responses are similar between treatments and genotypes indicating a water specific defect. For PER response, each independent trial consisted of ≥ 10 animals. (**g**) Water consumption of control (*UAS-DopR1* RNAi*/*  +), *elav-Gal4 > DopR1* RNAi and *MB247-Gal4* > *DopR1* RNAi flies. (**h**) Water consumption of control (*w* ^1118^), *DopR1* mutant (*elav-Gal4*/ + ; *dumb* ^2^/*dumb* ^2^) and rescue (*elav*-*Gal4* > *UAS-DopR1*; *dumb* ^2^/*dumb* ^2^ and *MB*2*47-Gal4 > UAS-DopR1*; *dumb* ^2^/*dumb* ^*2*^). All data represented mean ± S.E.M (n = 3–6). Student’s *t*-test or one-way ANOVA followed by Tukey’s *post hoc* test, **p < 0.01, ***p < 0.001; n.s., not significant.

To apply this system to further explore the neural regulation of thirst, we subjected flies to control fed and hydrated conditions, hydrated starvation, dehydration, and dehydrating starvation conditions for 6 hours. mRNA was then collected from fly heads and RNA sequencing was performed (Supplementary Table 1). We found 477 transcripts up-regulated specifically by dehydration. By GO terms from the molecular function ontology, 53 of these were either transmembrane transporter activity or signal transducer activity (Supplementary Table 2, Supplementary Figs 2 and 3). We selected 20 of these candidates to test for a role in regulating water consumption behaviour using a pan-neuronal (*elav-Gal4*) RNAi strategy^25^. We observed no major neuronal role in regulating drinking behaviour for *ALiX*, *brp*, *Ca-α1 T*, *msn*, *Ptp10D*, *rdgB*, *Shab*, *SK*, *slik*, *slo*, or *Stat92E* (Supplementary Fig. 1), however we did confirm multiple new negative regulators of water consumption (Supplementary Fig. 2). These include the voltage gated calcium channel *Ca-α1D*, the cell adhesion molecule *Dscam1*, the voltage-gated potassium channel *eag*, the transmembrane tyrosine phosphatase *Lar*, the voltage-gated sodium channel *para*, and the signalling molecule *Plc21C*. We also identified multiple positive regulators of drinking behaviour, including a GPCR of unknown function called *Calcium-independent receptor for α-latrotoxin* (*Cirl*), a Rho guanyl-nucleotide exchange factor *sif*, and the fly dopamine receptor *DopR1* (Fig. 4a, Supplementary Fig. 2m–p). Pharmacological studies have implicated the dopamine system in regulating thirst from dehydration in rats^26,27^, and more recently dopamine was implicated in water reward-based learning in flies^21^, so we investigated a role for *DopR1* in water consumption further.

RNA-seq data showed that *DopR1* was upregulated specifically by dehydration (Fig. 4a) and this was confirmed by qPCR (Fig. 4b). We next tested a *DopR1* mutant (*dumb* ^2^), which showed a strong decrease in water intake compared to control (Fig. 4c). This phenotype was specific for water consumption, since food intake was normal in these animals (Fig. 4d). Further, while dehydrated control animals exhibit a strong proboscis extension response (PER) to water, dehydrated *DopR1* mutants are much less sensitive (Fig. 4e). Both genotypes responded equally to a 5% sucrose solution indicating that *DopR1* is acting to specifically to regulate thirst. To directly test a role for dopamine in regulation of thirst, we fed flies either control food or food spiked with L-dopa. While L-dopa treatment enhanced thirst behaviour in wild type animals, *DopR1* mutant flies were resistant to this effect and again all animals showed comparable sucrose responses (Fig. 4f). We tested sensory neuron Gal4 lines previously implicated in water perception (*ppk28-Gal4*, *Nanchung-Gal4* and two *PoxN Gal4* lines) however, none of these neuron populations were required for water consumption (Supplementary Fig. 4). Thus, with the available tools, we cannot provide data for or against *DopR1* in directly regulating water responsive sensory neurons. Since *DopR1* has been shown to regulate water reward through actions in the mushroom body^21^, we tested a role for *DopR1* in indirectly regulating water consumption via the mushroom body. *DopR1* knockdown either pan-neuronally (*elav-Gal4*) or in the mushroom body (*MB247-Gal4*) reduced water intake to a similar extent as the whole body *dumb* ^2^ mutation (Fig. 4g) and transgenic re-introduction of *DopR1* either across the nervous system, or specifically within the mushroom body, was sufficient to rescue water intake (Fig. 4h).

**Figure 5:**
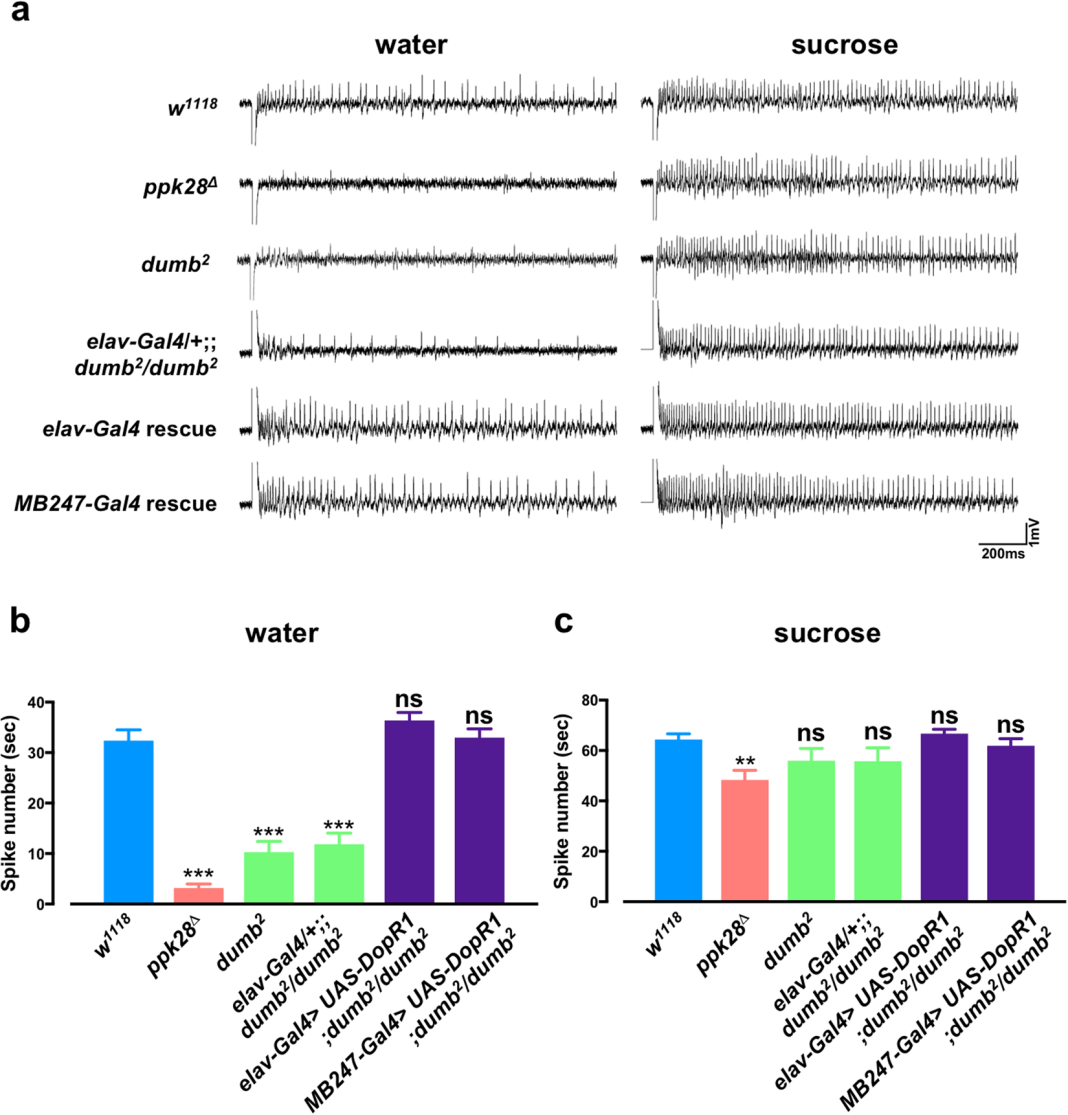
*DopR1* is required for water taste. Extracellular bristle recordings of *w* ^1118^ control, *ppk*2*8* mutant (*ppk*2*8* ^*Δ*^), *DopR1* mutant (*dumb* ^2^ and *elav-Gal4*/ + ;;*dumb* ^2^/*dumb* ^2^) and rescue (*elav*-*Gal4* > *UAS-DopR1*; *dumb* ^2^/*dumb* ^2^ *and MB247-Gal4 > UAS-DopR1*; *dumb* ^*2*^/*dumb* ^*2*^) flies after stimulation with water or 40 mM sucrose. (**a**) Representative spikes showing response to water (1 mM KCl) or 40 mM sucrose. (**b,c**) Mean total spike number for the first second of response to (**b**) water or (**c**) sucrose is shown. 3 to 5 L-type labellar bristles were recorded per animal. All data represent mean ± S.E.M (n = 9–17 animals). One-way ANOVA followed by Tukey’s *post hoc* test, **p < 0.01, ***p < 0.001; n.s., not significant.

Since *DopR1* was required for both drinking behaviour and water responsiveness by PER, we reasoned that *DopR1* may also play a role in regulating water taste perception. To test this, we performed electrophysiological recordings from water responsive taste sensilla on the *Drosophila* labellum. Control *w* ^1118^ males show a clear response to water and sucrose (Fig. 5a). In contrast *DopR1* mutant males showed a loss of water taste, and this phenotype was comparable to the water taste defect observed in the *ppk28* mutant flies. By temporal analysis we observe that the loss in water taste response in both *ppk28* and *DopR1* mutant animals is most prominent within the first second of stimulation (Supplementary Fig. 3), and this difference in spike frequency was significant (Fig. 5b) while again sucrose responses remain largely intact (Fig. 5c and Supplementary Fig. 3). Pan-neuronal (*elav-Gal4*) or mushroom body-specific (*MB247-Gal4*) re-introduction of *DopR1* also rescued the water taste response (Fig. 5a and b, Supplemtary Fig. 3) and both groups still exhibited intact responses to a sucrose solution. Thus, from an unbiased functional genome scan, we identify a role for *DopR1* in modulating water taste perception and water intake. Together, we describe a simple high throughput method of measuring water consumption, a process that is regulated by taste, food salinity, renal function, and other yet determined physiological parameters in the fly.

## Discussion

In this study, we describe a simple water consumption assay system that when combined with the power of fruit fly genetics will be useful to rapidly dissect multiple aspects of animal physiology through measuring water intake. This technique is highly reproducible, can be set up with minimal investment, and can be used to monitor water intake over several days. Importantly, known regulators of water taste and memory were also required in our system, meaning this assay can be used to identify factors that regulate water consumption at multiple levels. By coupling this system with transcriptomics and functional genomics, we demonstrate utility of this assay for screening and discovery of new genes that regulate water intake. Finally, we confirm a previously described role for dopamine in regulating water consumption and also implicate the dopamine system in regulating water taste, however the precise mechanisms for this regulation remain to be elucidated.

There are several aspects of our study that differ from previous work on this subject. First, we have developed a simple high throughput system that allows accurate measurement of water intake for a variety of purposes. This assay directly measures volume consumed and thus differs from previous systems evaluating time spent consuming water^17^ or assessment of water reward in memory^21^. In particular, we observed a strong increase in water consumption in animals that had consumed salty food, suggesting that this simple system could be applied to screen for molecular mechanisms governing hemolymph composition, renal function, or other whole animal physiological processes where water concentration is a factor. Through a synaptic silencing approach, we found *ppk28*, *nan* and multiple *PoxN-Gal4* expressing neurons were not necessary for water consumption behaviour (Supplementary Fig. 4). Highlighting that either there are additional yet unidentified water consumption neurons that are critical for water intake, or conversely that the function of known water taste neurons are not dependent on tetanus-sensitive synaptic output to mediate water intake.

We found both *ppk28* and *DopR1* were required for water intake in this system, and dehydration pre-treatment was not essential to observe water consumption differences between genotypes. This data is in accordance with previous work describing a role for *ppk28* in controlling time spent drinking, although this previous study required 18–24 hours of dehydration before drinking behaviour could be evaluated^17^. Similarly, in a water seeking assay, Lin *et al*. found that minimum 6 hours of dehydration was required for flies to begin seeking water^21^ and both water seeking and drinking behaviour was found to be independent of *ppk28* ^17^ or *DopR1* ^21^, while water learning required both. This result differs in some regard to the data we present here, where both *DopR1* and *ppk28* were found to be critical for naïve water consumption. This difference could be attributed to the complexity of our system, which should identify animals with defects in hygrosensation, water taste, thirst, learning and memory and animals that cannot regulate hemolymph osmolarity. Regardless, we present a new, simple and high throughput assay system to evaluate water consumption and this assay compliments the range of existing techniques.

*DopR1* has been implicated in water reward after dehydration, through actions on the γ-lobe of the mushroom body^21^. Our results here are in line with the overall findings by Lin *et al*. that *DopR1* acts in the mushroom body to regulate water intake. Additionally, we found that *DopR1*, through actions in the mushroom body, also indirectly regulates water perception or taste. We have also shown that *L-dopa* treatment can enhance wild type water response but not in *DopR1* mutants, suggesting that dopamine can play an immediate role in promoting water responses. However, more work is required before we can conclusively rule out a developmental role for DopR1 in the appropriate development of water neurons, and the temporal requirement for *DopR1* remains to be determined. Interestingly, in patients with Parkinson’s disease (PD), which involves a gradual decline in the dopamine system, a 40% decrease in daily water consumption has been reported^28–33^, and water intake inversely correlated with disease severity. Importantly, in PD patients decreased water intake precedes constipation or motor symptoms in some cases by decades^28^. Thus, dopamine may also regulate thirst and water taste in humans.

Together, we describe a simple high throughput assay system to investigate water consumption, and through testing this system we uncovered an unanticipated link between dopamine and the regulation of water taste, providing a potential molecular mechanism for why water palatability increases when an animal is thirsty.

## Methods

### Fly Strains

Drosophila melanogaster were grown on standard fly food containing sugar, yeast, molasses cornmeal, nipagin, agar and 0.04% propionic acid. All flies tested were 7 to 10-days-old males. *w* ^1118^ was used as wild type control. *UAS*-*DopR1*.RNAi (201154) and *w* ^1118^ were obtained from the Vienna Drosophila Resource Center. *MB247*-*Gal4*, 50742; *UAS-TeTxLC.TNT* (*UAS-TNT*, 28838) and *UAS-TeTxLC.IMP TNT* (*UAS-iTNT*, 28841); *UAS*-*ALiX*.RNAi, 50904; *UAS*-*brp*.RNAi, 25891; *UAS*-*Ca-α1D*, 25830; *UAS*-*Ca-α1T*.RNAi, 39029; *UAS*-*Cirl*.RNAi, 34821; *UAS*-*Dscam1*.RNAi, 38945; *UAS*-*eag*.RNAi, 31679; *UAS*-*Lar*.RNAi, 34965; *UAS*-*msn*.RNAi, 28791; *UAS*-*para*.RNAi, 33923; *UAS*-*Plc21C*.RNAi, 32438; *UAS*-*Ptp10D*.RNAi, 39001; *UAS*-*rdgB*.RNAi, 28796; *UAS*-*sif*.RNAi, 25789; *UAS*-*SK*.RNAi, 27238; *UAS*-*slik*.RNAi, 35179; *UAS*-*slo*.RNAi, 55405; *UAS*-*Stat92E*.RNAi, 35600; were obtained from the Bloomington Drosophila Stock Center. The *ppk28*-null mutant line (*ppk28* ^*Δ*^) was obtained from Kristin Scott’s lab^17^, *DopR1* mutant line (*dumb* ^2^) was obtained from Bruno Van Swinderen’s lab^34^ and *UAS-DopR1* transgene was obtained from Barry J. Dickson’s lab^35^. *elav-Gal4* with *UAS-Dicer2* on X chromosome was previous reported^25^.

### Water consumption assays

To measure water consumption, flies were tested in vials containing dehydrated food with 200 μl distilled water provided in a small PCR tube (Eppendorf). To make dehydrated food, 0.4 ml of food was placed in a vial and then desiccated for 2 weeks. Each experiment included an identical vial without flies to measure the evaporation rate. Water consumption was calculated by measuring the weight difference of PCR tube after correcting for the evaporation rate. For blue dye experiments, food colouring blue dye (1:2000; Queen Fine Foods Pty. Ltd., QLD Australia) solution was added in the PCR tube. For NaCl experiments, 1–5% NaCl (Sigma-Aldrich) was dissolved in the fly food and then desiccated for 2 weeks. Feeding assays are similar to water consumption assays except that flies were tested in an empty vial with wet kimwipes and 200 μl 5% sucrose solution was supplied to flies in the PCR tube.

### Proboscis extension response assays

For standard proboscis extension response (PER) assays, male flies were fed in vials and tested as described previously^36^, with slight modifications.

In brief, 10 experimental flies were glued to a glass slide to avoid their escape. The flies were starved at 19 °C and 70% humidity for 2 hours. To measure water and sugar response, each fly was tested with pure water or 5% sucrose (Sigma-Aldrich) stimuli two times. The percentage of PER was calculated as the sum of responses divided by the number of total tested flies (x 100%). For each genotype, 3 batches of 10 flies were tested on different days as independent triplicates. For L-dopa experiments, 3 mg/ml L-dopa precursor (Sigma-Aldrich) was dissolved in the fly food, and flies were maintained on this fly food for 2 days before PER assays. To measure water and sugar response, fixed flies were first starved for 1.5 hours (19 °C and 70% humidity).

### RNA Extraction and RNA Sequencing

Total RNA was extracted from fly heads by homogenization and cell lysis using TRIzol (LifeTechnologies) according to the manufacturer’s instructions. Further sample processing, library preparation and TruSeq RNA sequencing and analysis of the sample were performed by BGI (Shenzhen, China). Briefly, the total RNA samples were treated with DNase I to degrade any possible DNA contamination. mRNA was then enriched using oligo(dT) magnetic beads (for eukaryotes) and mixed with the fragmentation buffer and fragmented into short fragments (about 200 bp). The first strand of cDNA is synthesized by using a random hexamer-primer. Buffer, dNTPs, RNase H and DNA polymerase I are added to synthesize the second strand. The double strand cDNA is purified with magnetic beads. End reparation and 3′-end single nucleotide A (adenine) addition is then performed. Finally, sequencing adaptors are ligated to the fragments. The fragments are enriched by PCR amplification. During the QC step, Agilent 2100 Bioanaylzer and ABI StepOnePlus Real-Time PCR System are used to qualify and quantify of the sample library. The library products are ready for sequencing via Illumina HiSeq^TM^ 2000 or other sequencer when necessary.

### Reverse transcription quantitative real-time PCR (RT-qPCR)

Total RNA was extracted from fly heads by homogenization and cell lysis using TRIzol (LifeTechnologies) according to the manufacturer’s instructions. Single-stranded cDNA was synthesized from 2 µg total RNA according to the manufacturer’s procedure (LifeTechnologies). The primers used for SYBR Green RT-qPCR were as follows: for *DopR1*, sense, 5′-ACG ATG GCA CAA CGT TGA CA-3′ and antisense, 5′-GCA CCG ATA GGA AGA TGC CA-3′; for *18 S*, sense, 5′-TCT AGC AAT ATG AGA TTG AGC AAT AAG-3′ and antisense, 5′-AAT ACA CGT TGA TAC TTT CAT TGT AGC-3′. RT-qPCR was performed using the Applied Biosystems 7900HT Real-Time PCR System. Relative quantification of mRNA levels was performed using the comparative Cq method (∆∆Cq method) with *18 S* as the reference gene.

### Screening of differentially expressed genes (DEGs)

The *p* value was used to detect the difference in gene expression in two different samples. *p* value corresponds to differential gene expression test. False Discovery Rate (FDR) is a method to determine the threshold of *p*-value in multiple tests^37^. *p* value < 0.0001 and FDR ≤ 0.001 was used as the threshold to judge the significance of gene expression difference.

### Electrophysical recording

The electrophysiological responses of labellar taste neurons were recorded by using the tip recording method, similar to those described previously^38–40^. Briefly, all recordings were performed on L-type labellar bristles of 7–10 day-old male flies. 3–5 individual L-type bristles were recorded on each fly. The recording electrode (tip diameter, 10–12μm) was filled with 1 mM KCl for water sensing response or 40 mM sucrose in 30 mM tri-choline chloride (TCC; Sigma-Aldrich, as an electrolyte) for sucrose sensing response. Each bristle was first recorded for water response and then healthiness confirmed by checking response to sucrose. Signals were acquired using an AxonClamp 900 A amplifier and digitized with a 1400 A D-A converter (Molecular Devices) at a sampling rate of 10 kHz (filtered at 3 kHz). Electric signals were further amplified and filtered by a second amplifier (CyberAmp 320, Axon Instrument, Inc., USA, gain × 100, eighth order Bessel pass-band filter 1600Hz).

Data was analyzed using the Clampfit 10 software (Molecular Devices). Spikes between 0 and 2 s after initiation of stimuli were counted as firing frequency evoked by the tastant. The mean value of spikes was calculated on 3–5 bristles recorded on each fly as one statistical sample. The mean ± SEM in figures and text were based on number of flies.

### Data analysis

All the experiments were performed at least three times. All values are expressed as mean ± SEM. Data was analyzed by Student’s *t*-test or one-way ANOVA followed by Tukey’s *post hoc* test using GraphPad Prism 6 (GraphPad Software, San Diego, CA). *p* < 0.05 was considered statistically significant.

## Electronic supplementary material

Supplementary material and figures

Supplementary Table 1

Supplementary Table 2
