# Supplementary material for: A simple high throughput assay to evaluate water consumption in the fruit fly
Source: Sci Rep. 2017 Dec 1;7:16786. doi: 10.1038/s41598-017-16849-6 (PMC5711950; doi:10.1038/s41598-017-16849-6)
Supplement: Supplementary file 1 — Supplementary material and figures [file 41598_2017_16849_MOESM1_ESM.pdf]

A simple high throughput assay to evaluate water consumption in the fruit fly

Man-Tat Lau[1, 2], Yong Qi Lin[1, 2], Stefan Kisling[2], James Cotterell[1, 2], Yana A. Wilson[1, 2], Qiao-Ping Wang[1, 2], Thang M. Khuong[1, 2], Noman Bakhshi[1, 2], Tiffany A. Cole[1, 2], Lisa J. Oyston[1, 2], Adam R. Cole[1,3], G. Gregory Neely[1, 2]

[1]The Dr. John and Anne Chong Lab for Functional Genomics, Charles Perkins Centre and School of Life & Environmental Sciences, The University of Sydney NSW 2006, Australia

[2]Neuroscience Division, Garvan Institute of Medical Research, 384 Victoria Street, Darlinghurst, Sydney, NSW 2010, Australia.

[3]Sacred Heart College, Retreat Rd, Newtown, Geelong, Victoria 3220, Australia

SUPPLEMENTAL FIGURE LEGENDS

Supplemental Figure 1. Dehydration response genes that do not regulate water consumption. Water consumption for control (*UAS*-RNAi flies *x* w^{1118}) and candidate *elav-Gal4>*RNAi flies. All data presented as mean ± S.E.M (n=3-6). Student's *t*-test, not significant.

Supplemental Figure 2. Dehydration response genes that regulate water consumption. Water consumption assay of control (*UAS*-RNAi flies *x* w^{1118}) and candidate *elav-Gal4>*RNAi flies. All data presented as mean ± S.E.M (n=3-6). Student's *t*-test, n.s., *, p <0.05; **, p <0.01.

Supplemental Figure 3. Extracellular bristle recordings of w^{1118} control, *ppk28* mutant (*ppk28*$^{\Delta}$), *DopR1* mutant (*dumb*^{2} and *elav-Gal4*/+;;*dumb*^{2}/*dumb*^{2}) and rescue (*elav-Gal4>UAS-DopR1*; *dumb*^{2}/*dumb*^{2} *and MB247-Gal4>UAS-DopR1*; *dumb*^{2}/*dumb*^{2}) flies after stimulation with water (1mM KCl) or 40mM sucrose. Mean spike number in response to water or sucrose is shown every 200 ms for a 2 sec. 3 to 5 L-type labellar bristles were recorded per animal. All data presented as mean ± S.E.M (n=9-17 animals).

Supplemental Figure 4. Blocking synaptic output in varius peripheral neuron do not regulate water consumption. Water consumption assay for control (*Gal4* flies *x iTNT*; inactive tetanus toxin) and active *TNT*-expressing flies (*Gal4* flies x *TNT*). All data presented as mean ± S.E.M (n=3-6). Student's *t*-test, n.s., not significant.

Supplemental Table 1. (a) All differential expressed genes in Fed Vs Dehydration conditions. **(b)** All differential expressed genes in Fed Vs Hydrated starvation conditions. **(c)** All differential expressed genes in Fed Vs Dehydrated starvation conditions.

Supplemental Table 2. The differential up-regulated genes in Dehydration conditions belong to GO: transmembrane proteins or signalling molecules

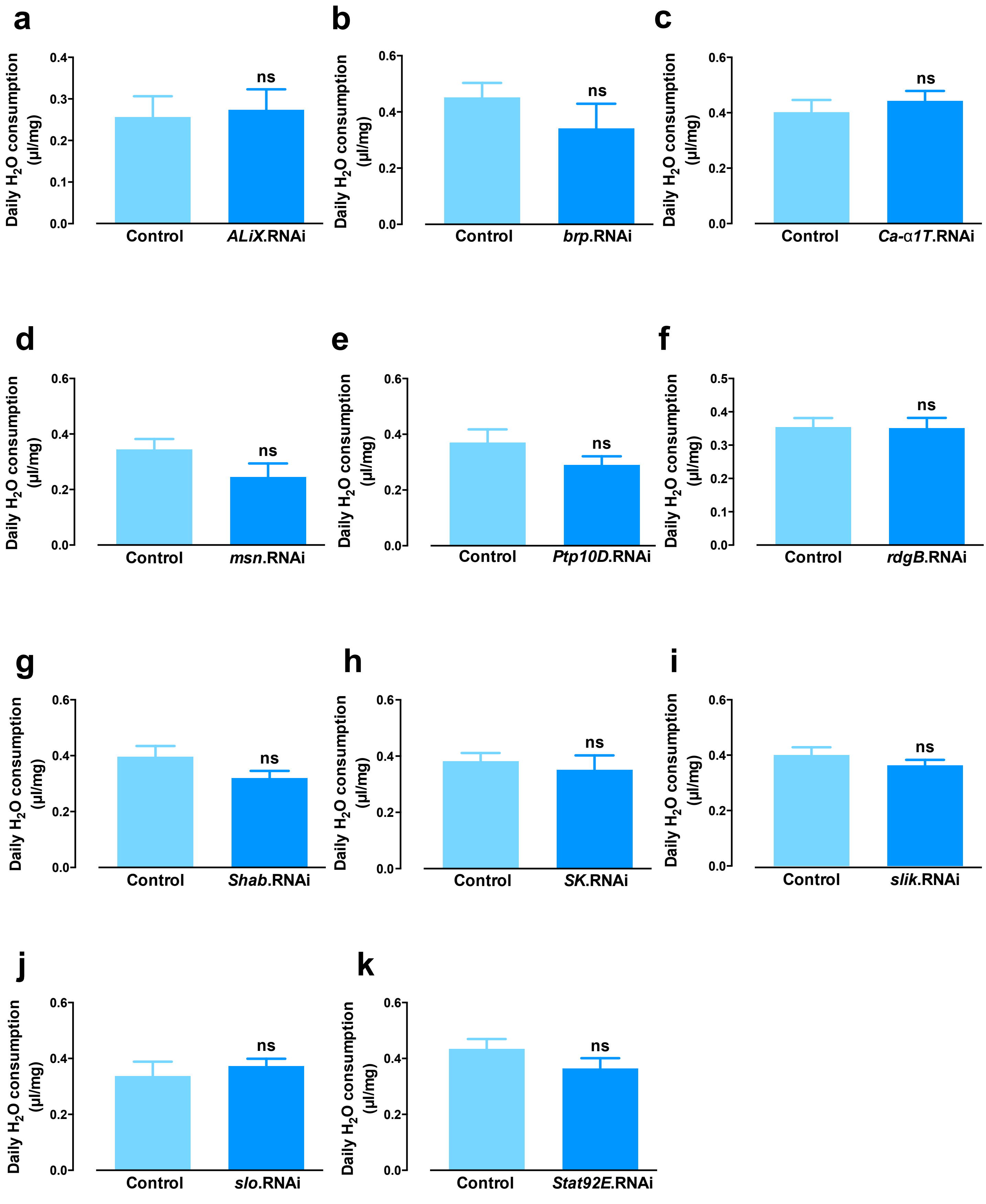

Supplementary Figure 1

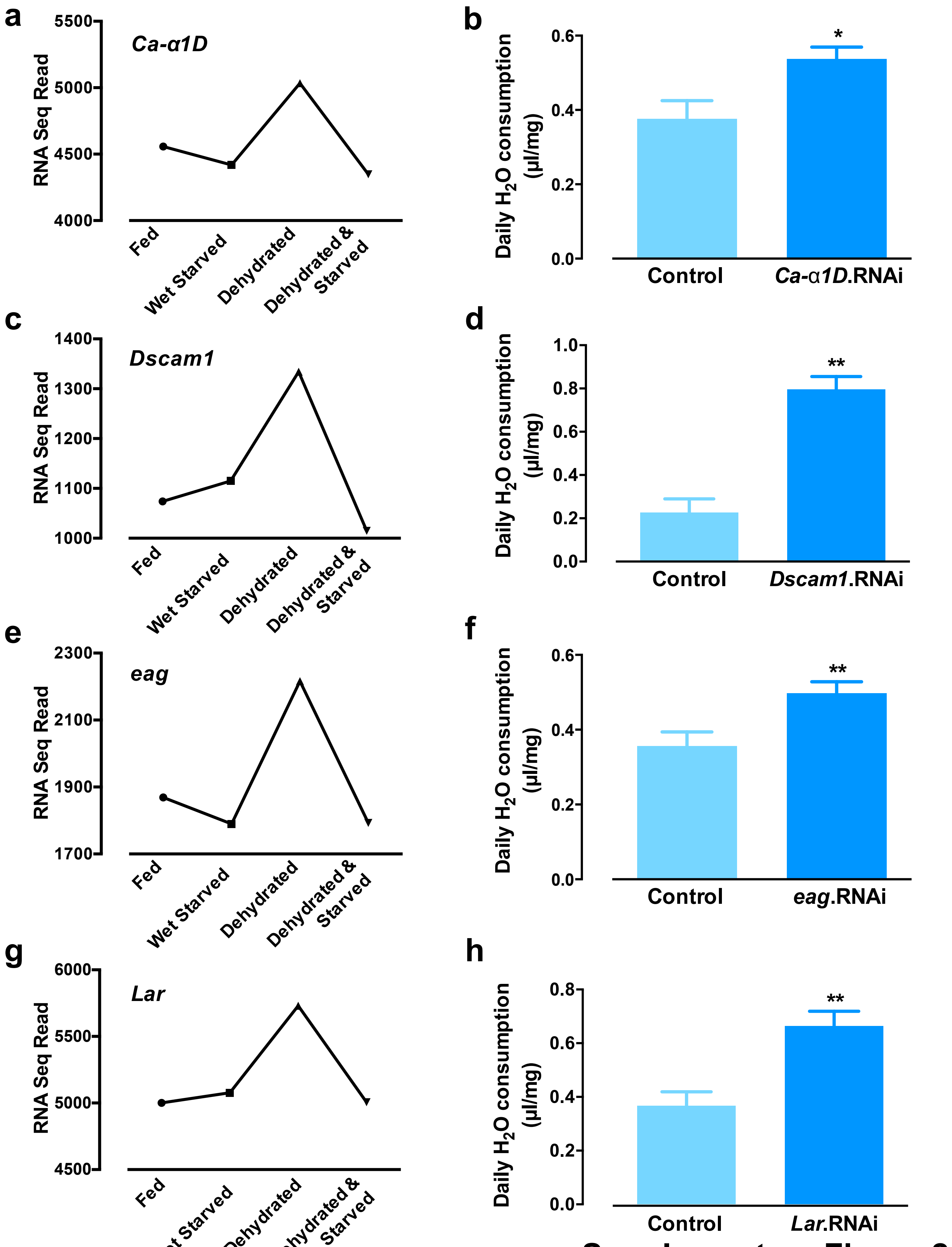

Supplementary Figure 2

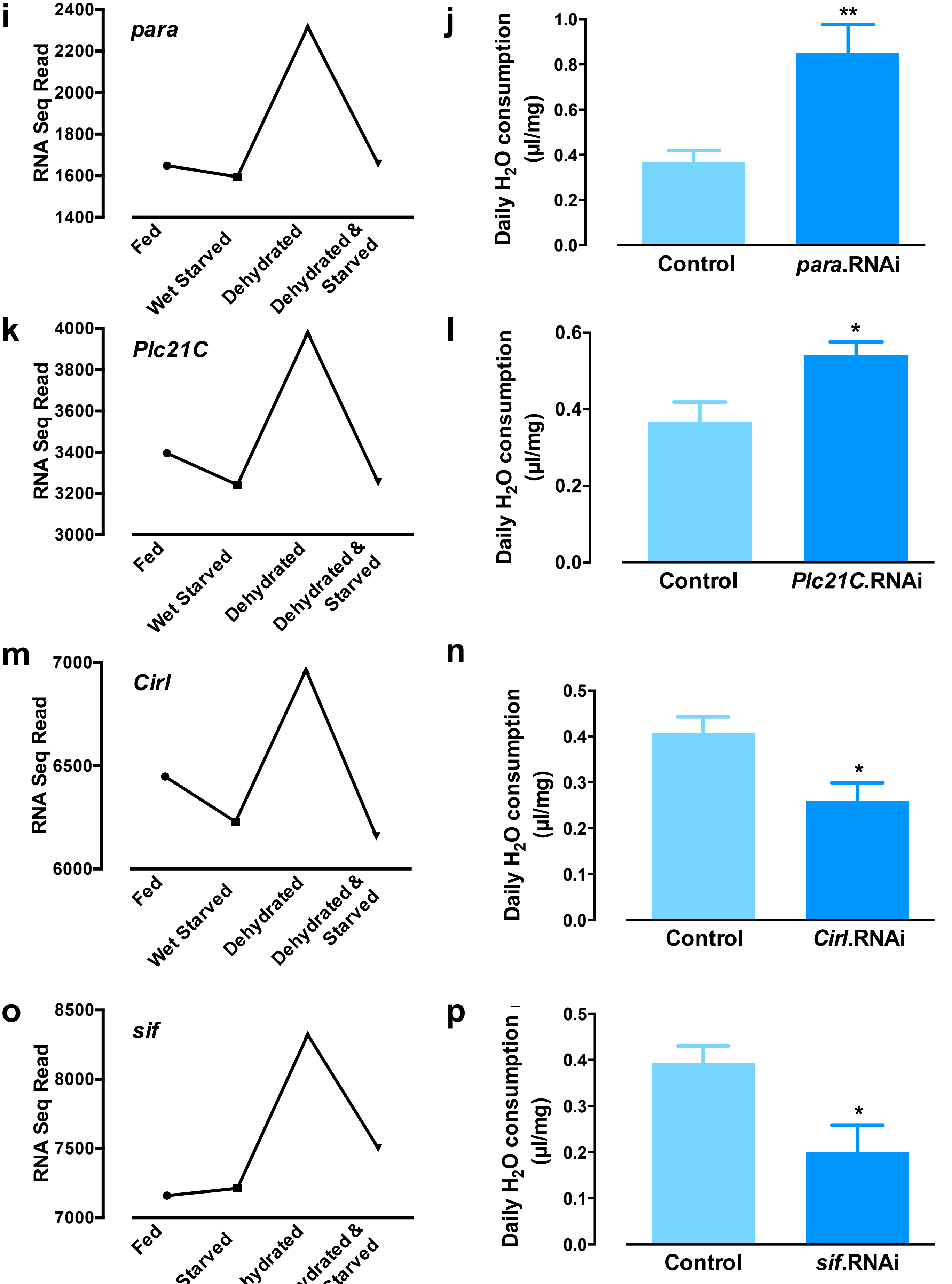

Supplementary Figure 2

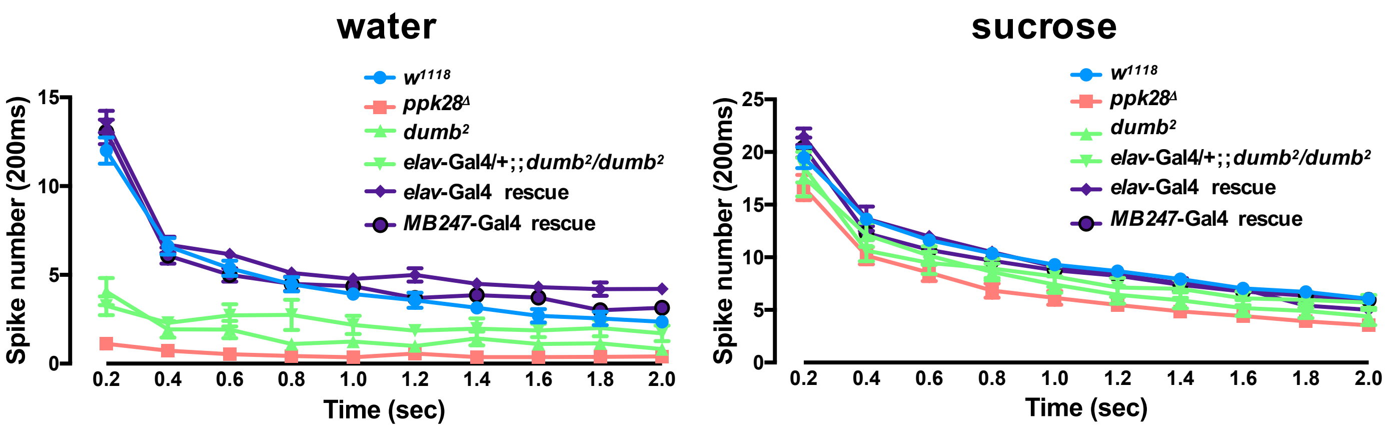

Supplementary Figure 3

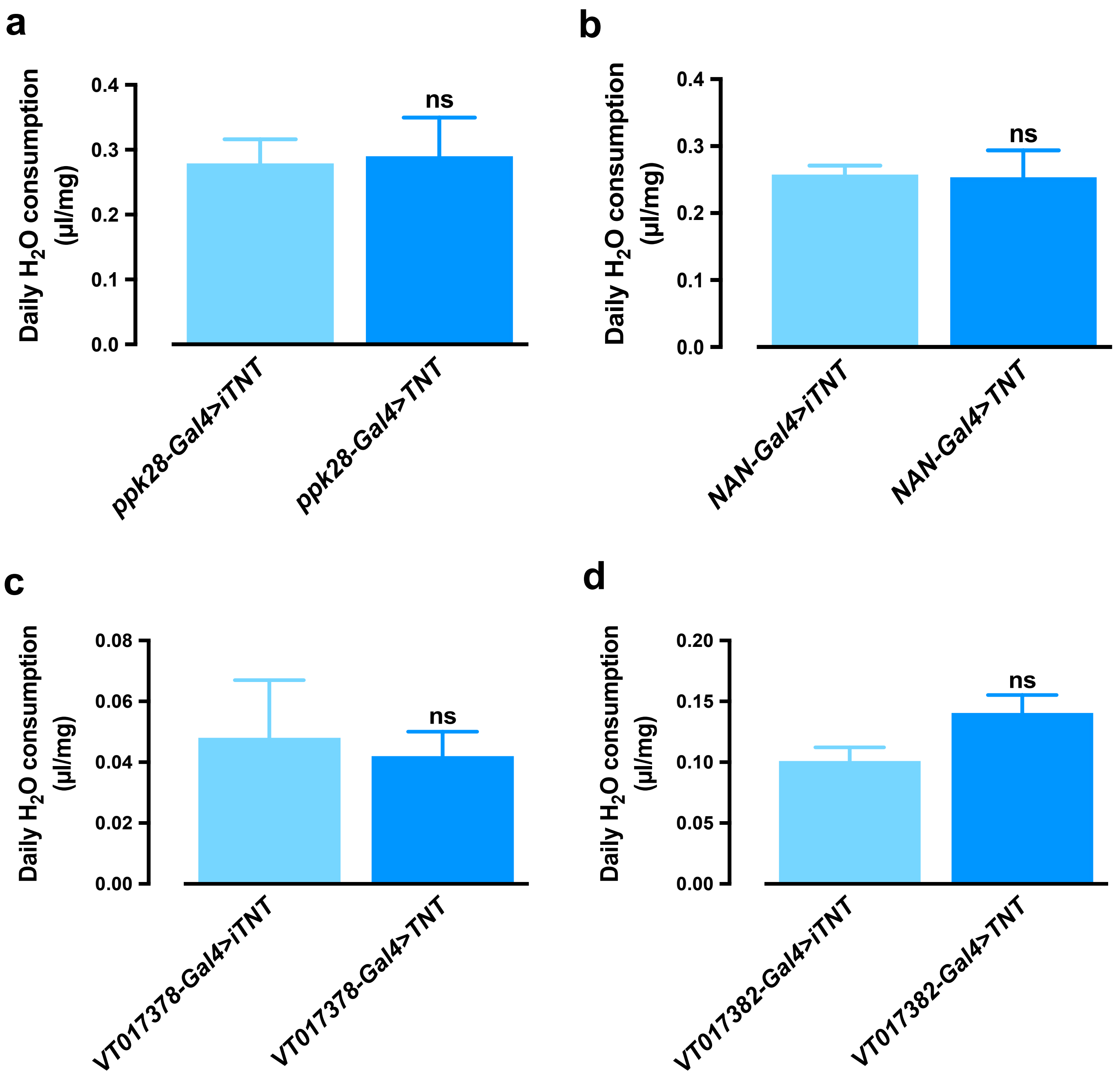

Supplementary Figure 4
